# Association between hypertriglyceridemic-waist phenotype and cardiovascular disease: A cohort study and meta-analysis

**DOI:** 10.3389/fcvm.2022.940168

**Published:** 2022-08-04

**Authors:** Xiaowei Zheng, Xiao Ren, Minglan Jiang, Longyang Han

**Affiliations:** Department of Public Health and Preventive Medicine, Wuxi School of Medicine Jiangnan University, Wuxi, China

**Keywords:** hypertriglyceridemic, cardiovascular risk, cardiovascular disease, metabolism, obesity

## Abstract

**Background:**

The association between hypertriglyceridemic-waist (HTGW) phenotype and cardiovascular disease (CVD) remains inconsistent and debatable. We aimed to prospectively investigate the relationship between HTGW phenotype and CVD.

**Methods and results:**

We included 8,216 participants from the China Health and Retirement Longitudinal Study. Participants were categorized into four subgroups: NTNW: normal triglyceride levels and normal waist circumference; HTNW: high triglyceride levels and normal waist circumference; NTGW: normal triglyceride levels with enlarged waist circumference; HTGW: high triglyceride levels and enlarged waist circumference. A Cox proportional hazards model was applied to determine the association between HTGW phenotype and CVD. A meta-analysis was conducted to incorporate the results of the current study and the previous-related studies on the association of HTGW phenotype and CVD. In the present cohort study, compared to the NTNW phenotype, those with NTGW (Hazard ratios (*HRs*) 1.34, 95% confidence intervals (*CIs*) 1.16–1.55) and HTGW (*HRs* 1.37, 95% *CIs* 1.16–1.62) phenotype were significantly associated with CVD risk. The meta-analysis further confirmed the significant association between HTGW phenotype and CVD [the pooled relative risk for HTGW vs. NTNW was 1.39 (1.29–1.49)].

**Conclusion:**

The HTGW phenotype was associated with the increased risk of CVD, independently of established risk factors. A simple assessment of HTGW phenotypes might help to identify individuals with a high risk of developing CVD.

## Introduction

Cardiovascular disease (CVD), which includes ischemic cardiopathies and cerebrovascular diseases, is the largest single contributor to global mortality and is responsible for 18.6 million deaths in 2019, which was estimated to account for 32.3% of all-cause global deaths (1–3). Obesity, one of the well-known risk factors, is known to be associated with a higher risk of cardiovascular morbidity and mortality (4, 5). Thus, the management of obesity such as lifestyle modification, pharmacotherapy, and bariatric procedures, is significantly associated with weight loss and improvements in the CVD risk factors (6).

Body mass index (BMI) is the most commonly used index to measure overall body fat and for clinical diagnosis of obesity (7, 8). In recent years, visceral fat, rather than subcutaneous adiposity, has been proposed to more accurately reflect the fat distribution and metabolic changes, which may be a better predictor of CVD than BMI (9–11). As high-sensitive detection methods for visceral fat, computed tomography, and magnetic resonance imaging are often hard to achieve in a large-scale general population due to cost, time consumption, and radiation exposure (12). Waist circumference (WC) and waist–hip ratio are available for the measurement of visceral fat but cannot distinguish visceral adipose from subcutaneous abdominal fat (13). The hypertriglyceridemic-waist (HTGW) phenotype (the combination of an increased WC and hypertriglyceridemia), combined with the potential predictive value of WC and triglycerides (TG), is a simple and inexpensive tool for clinicians to identify individuals who have the greatest amount of visceral fat (14) and was reported associated with atherogenic and diabetogenic risk factors (15, 16), coronary artery disease (17), and stroke (18, 19). However, findings on the association between HTGW phenotype and CVD are not consistent. Several studies (20–22) but not all studies (23, 24) have reported a significant association between HTGW and CVD. In addition, results from different research designs and study populations limit the credibility.

To better understand and further extend our knowledge on the association between HTGW phenotype and CVD, we prospectively investigate whether HTGW phenotype was associated with the development of CVD based on the data from the China Health and Retirement Longitudinal Study (CHARLS). Furthermore, we conducted a meta-analysis that combined our current study with other previously published studies to assess overall evidence of the predictive effect of HTGW phenotype on CVD.

## Materials and methods

### Study population

China Health and Retirement Longitudinal Study is an ongoing nationally representative study that uses a multistage clustering sample method to select participants in China (25, 26). In brief, a total of 17,708 participants from 10,257 households recruited from 28 provinces within China were included at baseline (2011–2012, Wave 1). CHARLS respondents were followed up every 2 years, using a face-to-face computer-assisted personal interview. Three subsequent follow-ups were carried out in 2013–2014 (Wave 2), 2015–2016 (Wave 3), and 2017–2018 (Wave 4) among survivors. The ethics application for collecting data on human subjects in CHARLS was approved by the Biomedical Ethics Review Committee of Peking University (IRB00001052-11015), and all CHARLS participants provided written informed consent. The details of the CHARLS data are available on its website^1^.

**FIGURE 1:**
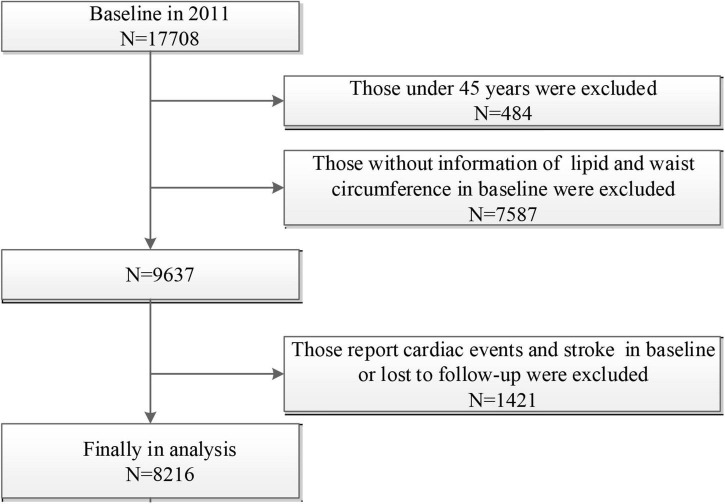
Flow chart of sample selection and the exclusion criteria.

In the present study, we excluded participants aged < 45 years old or without age information (*n* = 484), participants without information of lipid and WC at baseline (*n* = 7587), and those who report cardiac events and stroke at baseline or lost to follow-up (*n* = 1421). A final sample of 8,216 participants was included in this analysis (Figure 1).

### Sample collection, the definition of hypertriglyceridemic-waist (HTGW) phenotype, and cardiovascular disease (CVD)

Trained staff collected the venous blood samples in overnight fasting participants. Then, the samples were transported to the local laboratory timely and stored at 4°C. The blood samples were centrifuged and stored at −20°C before being transported to the central laboratory in Beijing and frozen at −80°C before analysis. All study laboratories had standardized certification. The enzymatic colorimetric test method was used to measure TG, total cholesterol (TC), high-density lipoprotein cholesterol (HDL-C), and low-density lipoprotein cholesterol (LDL-C). WC was measured with a soft tape at the level of the navel when participants were asked to hold their breath at the end of exhaling.

On the basis of the recommended criteria for HTGW phenotype (14, 27): a normal WC was less than 90 cm for men and less than 85 cm for women, and a normal TG level was less than 2.0 mmol/L for men and less than 1.5 mmol/L for women. Participants were divided into four groups: normal TG levels and normal WC (NTNW), high TG levels and normal WC (HTNW), normal TG levels and enlarged WC (NTGW), and high TG levels and enlarged WC (HTGW).

The primary outcome of the present study was incident CVD (stroke or cardiac events), and the secondary outcomes were stroke and cardiac events, separately. The incident of stroke or cardiac events was defined as new events that occurred from Wave 2 to Wave 4, based on a self-reported physician’s diagnosis [“Has a doctor ever told you that you had any heart disease (such as myocardial infarction, coronary heart disease, angina, congestive heart failure, or other heart problems) or stroke?”], following previously reported studies in CHARLS (28, 29).

### Covariates assessments

The covariates were collected at baselines such as age, sex, place of residence (rural vs. urban), smoking status (ever smoking vs. never smoking), educational level (illiteracy, primary school, middle school, high school, or above), drinking status (ever drinking vs. never drinking), BMI (the weight in kilograms divided by the square of the height in meters), and the presence or absence of other chronic diseases (hypertension, dyslipidemia, diabetes, cancer, chronic lung disease, kidney disease, liver disease, arthritis, digestive disease, and asthma). “Ever smoking” means that the respondent reported smoking at some point, and “never smoking” means that the respondent reported never having smoked. “Ever drinking” means that the respondent reports having had an alcoholic beverage in the past, and “never drinking” means that the respondent reported not having any alcoholic beverage in the past. Blood pressure was measured with an electronic sphygmomanometer (Omron HEM-7200 Monitor) after 5 min of rest in the sitting position and was defined as the average of three separate measurements. Hypertension was defined as systolic blood pressure ≥ 140 mm Hg, diastolic blood pressure ≥ 90 mm Hg, current use of antihypertensive medications, or self-reported history of hypertension. Diabetes was defined as fasting glucose ≥ 126 mg/dl, glycosylated hemoglobin (HbA1c) ≥ 6.5%, treatment for diabetes mellitus, or self-reported history of diabetes. Respondents completed a questionnaire where they reported weekly physical activity in three predefined categories (vigorous physical activity, moderate physical activity, and light physical activity).

### Statistical analysis

Participants’ baseline characteristics are presented as percentages for categorical variables, as the means with SD + for normally distributed variables and as medians with interquartile range for non-normally distributed variables. Demographic and clinical characteristics were compared between four groups by ANOVA or Kruskal–Wallis test for continuous variables and χ2 test for categorical variables. A Cox proportional hazards model was applied to calculate the hazard ratios (*HRs*) and 95% confidence intervals (*CIs*) for incident CVD, stroke, and cardiac events before and after adjusting for covariates. Potential covariates, such as age, sex, place of residence, education level, smoking, drinking, systolic blood pressure, physical activity, chronic diseases (dyslipidemia, diabetes mellitus, chronic lung disease, and stroke), and medications (anti-hypertensive, anti-dyslipidemic, and anti-diabetic) were included in the multivariable models.

Subgroup analyses were further performed to evaluate the association between HTGW phenotype and the risk of CVD according to sex, age, place of residence, BMI, smoking, drinking, hypertension, and education level subgroups. Two-tailed *p* < 0.05 was considered to be statistically significant. All statistical analyses were conducted using SAS statistical software (version 9.4, Cary, NC, United States).

### Meta-analysis

The current meta-analysis was conducted according to the recommendations of the Preferred Reporting Items for Systematic Review and Meta-Analysis (PRISMA) guidelines (30). In brief, Embase, PubMed, Medline, and Web of Science were searched from dataset inception up to 1 April 2022 for papers that have assessed the association between HTGW phenotype and risk of CVD. The search strategy combined terms related to HTGW and CVD without any restrictions on language or study type (details in Supplementary Methods). Literature search and data extraction were independently performed by 2 investigators. The following data elements were extracted from each included study: first author, year and place of study, study design, sample size, male proportion, WC and TG cut-off, study outcome, study quality, adjusted covariates, and effect size (*HRs*, *ORs*, or relative risks). The pooled RRs were used to assess the association between HTGW phenotype and the risk of CVD in the meta-analysis. Heterogeneity was assessed using Cochrane’s *Q* test and *I*^2^ statistic. Potential publication bias was examined using a funnel plot, Begg test, and Egger’s asymmetry test. All analyses were performed using STATA14.0 (Stata Corp LP, College Station, TX, United States). Two-tailed *p* < 0.05 was considered to be statistically significant. Detailed methods of this meta-analysis are described in Data s1.

## Results

### Baseline characteristics of study participants

In the present study, 8,216 participants (3,894 men and 4,322 women) were included in the analysis, and the average age was 58.92 ± 9.32 years. Most baseline characteristics were well balanced between the included and excluded participants, indicating that those enrolled participants basically represent the total participants of CHARLS (Supplementary Table 1). The prevalences of NTNW, NTGW, HTNW, and HTGW phenotypes were 49.05, 11.08, 23.94, and 15.93%, respectively. Table 1 shows the baseline characteristics of participants according to different HTGW phenotypes. Baseline characteristics, such as age, sex, living place, history of hypertension, dyslipidemia and diabetes mellitus, smoking, drinking, BMI, TG, TC, LDL-C, HDL-C, FBG, SBP, and DBP were significantly different among the four subgroups.

**TABLE 1:** Baseline characteristics of the study participants according to high triglyceride levels and enlarged waist circumference (HTGW) phenotype in baseline.

Variable	NTNW	HTNW	NTGW	HTGW	*P* value
No. of subjects	4030	910	1967	1309	
Age, years	59.46 ± 9.61	57.87 ± 8.91	58.69 ± 9.29	58.33 ± 9.11	<0.001
Sex, n (%)					<0.001
Male	2474 (61.39)	329 (36.15)	772 (39.25)	319 (24.37)	
Female	1556 (38.61)	581 (63.85)	1195 (60.75)	990 (76.53)	
Living place, n (%)					<0.001
Urban	1141 (28.31)	298 (32.75)	798 (40.57)	550 (42.02)	
Rural	2889 (71.69)	612 (67.25)	1169 (59.43)	759 (57.98)	
Education level, n (%)					0.104
Illiteracy	1129 (28.01)	310 (34.07)	597 (30.35)	441 (33.69)	
Primary school	1755 (43.55)	346 (38.02)	744 (37.82)	501 (38.273)	
Middle school	768 (19.06)	172 (18.90)	419 (21.30)	244 (18.64)	
High school or above	378 (9.38)	82 (9.01)	207 (10.52)	123 (9.40)	
**Medical history**					
Hypertension, n (%)	823 (20.42)	178 (19.56)	445 (22.62)	314 (23.99)	0.003
Dyslipidemia, n (%)	150 (3.72)	67 (7.36)	194 (9.86)	211 (16.12)	<0.001
Diabetes mellitus, n (%)	120 (2.98)	38 (4.18)	139 (7.07)	148 (11.31)	<0.001
Smoking, n (%)	2022 (50.17)	295 (32.42)	615 (31.27)	309 (23.61)	<0.001
Drinking, n (%)	1859 (46.13)	312 (34.29)	724 (36.81)	371 (28.34)	<0.001
BMI, kg/m^2^	21.25 (19.57–22.95)	22.13 (20.40–23.56)	25.66 (23.91–27.42)	26.42 (24.54–28.52)	<0.001
TG, mg/dL	81.42 (63.72–106.20)	198.24 (159.30–257.54)	96.45 (74.34–117.71)	202.67 (162.84–273.47)	<0.001
TC, mg/dL	184.02 (160.83–207.22)	203.54 (179.00–232.73)	189.05 (167.78–212.24)	206.06 (177.84–234.28)	<0.001
LDL-C, mg/dL	110.57 (91.24–131.44)	113.27 (89.30–139.56)	120.62 (100.90–141.11)	114.82 (88.53–144.20)	<0.001
HDL-C, mg/dL	55.28 (46.78–65.72)	42.91 (25.57–51.42)	49.10 (41.75–58.76)	39.05 (33.25–46.78)	<0.001
FBG, mg/dL	99.72 (92.16–108.54)	106.92 (97.56–119.61)	102.24 (94.86–111.60)	109.71 (99.54–126.90)	<0.001
SBP, mmHg	126.61 ± 20.66	128.57 ± 20.53	133.35 ± 21.72	135.01 ± 20.14	<0.001
DBP, mmHg	73.76 ± 11.32	75.70 ± 12.10	77.38 ± 11.68	78.31 ± 11.27	<0.001

BMI, body mass index; TG, triacylglycerol; TC, total cholesterol; LDL-C, low-density lipoprotein-cholesterol; HDL-C, High-density lipoprotein-cholesterol; FBG, fasting blood glucose; SBP, systolic blood pressure; DBP, diastolic blood pressure; NTNW, normal triglyceride levels and normal waist circumference; HTNW, high triglyceride levels and normal waist circumference; NTGW, normal triglyceride levels with enlarged waist circumference; HTGW, high triglyceride levels and enlarged waist circumference.

Continuous variables are expressed as mean ± standard deviation, or as median (interquartile range). Categorical variables are expressed as frequency (percent).

Normal WC was less than 90 cm for men and less than 85 cm for women, and a normal triglyceride level was less than 2.0 mmol/L for men and less than 1.5 mmol/L for women.

### Association between hypertriglyceridemic-waist (HTGW) phenotypes and cardiovascular disease (CVD), stroke, and cardiac events

After 6 years of follow-up (Wave 2 to Wave 4), a total of 1,471 respondents experienced CVD (such as 491 strokes and 1,108 cardiac events). The event rates of CVD, stroke, and cardiac events were significantly higher among patients with HTGW phenotype (Table 2). Compared to individuals with NTNW phenotype, those with NTGW (*HRs* 1.76, 95% *CIs* 1.53–2.02) and HTGW (*HRs* 1.90, 95% *CIs* 1.62–2.21) phenotype had significantly increased risk of CVD in the crude model. After adjustment for age, sex, and other variables, individuals with NTGW (*HRs* 1.34, 95% *CIs* 1.16–1.55) and HTGW (*HRs* 1.37, 95% *CIs* 1.16–1.62) phenotypes were still significantly associated with the higher risk of CVD. Similarly, individuals with NTGW and HTGW phenotypes were significantly associated with an increased risk of strokes and cardiac events (Table 2).

**TABLE 2:** Risk of cardiovascular disease (CVD), stroke and cardiac events by high triglyceride levels and enlarged waist circumference (HTGW) phenotype.

Variable	NTNW	HTNW	NTGW	HTGW	*P* trend
**CVD** ^†^					
Case, n(%)	567 (14.07)	154 (16.92)	440 (22.37)	310 (23.68)	
Unadjusted	1.00 (Ref)	1.24 (1.02–1.51)	1.76 (1.53–2.02)	1.90 (1.62–2.21)	<0.001
Age and sex- adjusted	1.00 (Ref)	1.25 (1.02–1.52)	1.75 (1.52–2.01)	1.86 (1.58–2.19)	0.025
Multivariable-adjusted*	1.00 (Ref)	1.16 (0.97–1.39)	1.34 (1.16–1.55)	1.37 (1.16–1.62)	<0.001
**Stroke**					
Case, n(%)	183 (4.54)	48 (5.27)	150 (7.63)	110 (8.40)	
Unadjusted	1.00 (Ref)	1.17 (0.85–1.61)	1.69 (1.36–2.10)	1.88 (1.49–2.39)	<0.001
Age and sex- adjusted	1.00 (Ref)	1.34 (0.97–1.85)	1.86 (1.50–2.32)	2.23 (1.24–1.99)	<0.001
Multivariable-adjusted*	1.00 (Ref)	1.26 (0.92–1.75)	1.52 (1.18–1.96)	1.72 (1.29–2.30)	<0.001
**Cardiac events**					
Case, n(%)	430 (10.67)	122 (13.41)	324 (16.47)	232 (17.72)	
Unadjusted	1.00 (Ref)	1.28 (1.05–1.57)	1.59 (1.38–1.84)	1.72 (1.49–2.02)	<0.001
Age and sex- adjusted	1.00 (Ref)	1.22 (0.99–1.50)	1.51 (1.30–1.74)	1.57 (1.33–1.86)	<0.001
Multivariable-adjusted*	1.00 (Ref)	1.19 (0.97–1.46)	1.46 (1.25–1.69)	1.52 (1.28–1.80)	<0.001

^†^CVD including stroke and cardiac events.

*Multivariable-adjusted for age, sex, place of residence, education level, smoking, drinking, systolic blood pressure, physical activity, chronic diseases (dyslipidemia, diabetes mellitus, chronic lung disease and stroke) and medications (anti-hypertensive, anti-dyslipidemic and anti-diabetic).

In the subgroup analysis, significant associations of HTNW and HTGW phenotype with risk of CVD were observed in almost all subgroups, and significant interactions were observed between HTGW phenotype and age (*p* < 0.001), BMI (*p* < 0.001), and education level (*p* = 0.013) in relation to CVD (Table 3).

**TABLE 3:** Subgroup analysis of the association between high triglyceride levels and enlarged waist circumference (HTGW) phenotype and risk of cardiovascular disease (CVD).

Characteristics	NTNW	HTNW	NTGW	HTGW	*P* value	*P-interaction*
**Sex**						
Male	1.00 (Ref)	1.13 (0.83–1.53)	1.39 (1.11–1.73)	1.60 (1.20–2.13)	<0.001	0.264
Female	1.00 (Ref)	1.15 (0.91–1.45)	1.28 (1.06–1.56)	1.28 (1.04–1.58)	0.013	
**Age, years**						
< 60	1.00 (Ref)	1.15(0.89–1.48)	1.44(1.17–1.76)	1.31(1.04–1.65)	<0.001	<0.001
≥ 60	1.00 (Ref)	1.17 (0.90–1.51)	1.25 (1.01–1.54)	1.41 (1.11–1.79)	0.005	
**Place of residence**						
Urban	1.00 (Ref)	1.13 (0.91–1.41)	1.30 (1.08–1.57)	1.39 (1.12–1.72)	0.001	0.859
Rural	1.00 (Ref)	1.24 (0.90–1.72)	1.48 (1.17–1.88)	1.39 (1.06–1.82)	0.005	
**BMI, kg/m^2^**						
< 24	1.00 (Ref)	1.18 (0.96–1.45)	1.23 (0.98–1.54)	1.49 (1.12–1.98)	0.003	<0.001
≥ 24	1.00 (Ref)	0.99 (0.65–1.52)	1.35 (1.04–1.73)	1.32 (1.01–1.72)	0.030	
**Smoking**						
No	1.00 (Ref)	1.19 (0.95–1.50)	1.294 (1.07–1.55)	1.33 (1.08–1.62)	0.004	0.153
Yes	1.00 (Ref)	1.11 (0.81–1.51)	1.44 (1.13–1.82)	1.48 (1.09–2.01)	0.002	
**Drinking**						
No	1.00 (Ref)	1.23 (0.98–1.54)	1.35 (1.12–1.63)	1.35 (1.09–1.66)	0.003	0.883
Yes	1.00 (Ref)	1.07 (0.79–1.46)	1.32 (1.05–1.67)	1.42 (1.07–1.89)	0.006	
**Hypertension**						
No	1.00 (Ref)	1.18 (0.96–1.46)	1.39 (1.18–1.65)	1.44 (1.18–1.74)	<0.001	0.725
Yes	1.00 (Ref)	1.09 (0.75–1.57)	1.17 (0.87–1.56)	1.14 (0.82–1.59)	0.350	
**Education level, n (%)**						
Below primary school	1.00 (Ref)	1.14 (0.84–1.54)	1.10 (0.85–1.44)	1.29 (0.97–1.73)	0.107	0.013
Primary school	1.00 (Ref)	1.08 (0.79–1.48)	1.47 (1.09–2.09)	1.75 (1.27–2.41)	0.002	
Middle school	1.00 (Ref)	1.15 (0.73–1.82)	1.52 (1.10–2.10)	1.03 (0.69–1.56)	0.280	
High school or above	1.00 (Ref)	1.30 (0.76–2.20)	1.21 (0.80–1.82)	1.41 (0.87–2.28)	0.171	

In the multivariate models, confounding factors such as age, sex, place of residence, education level, smoking, drinking, systolic blood pressure, physical activity, chronic diseases (dyslipidemia, diabetes mellitus, chronic lung disease and stroke) and medications (anti-hypertensive, anti-dyslipidemic and anti-diabetic) were included unless the variable was used as a subgroup variable.

### Meta-analysis

**FIGURE 2:**
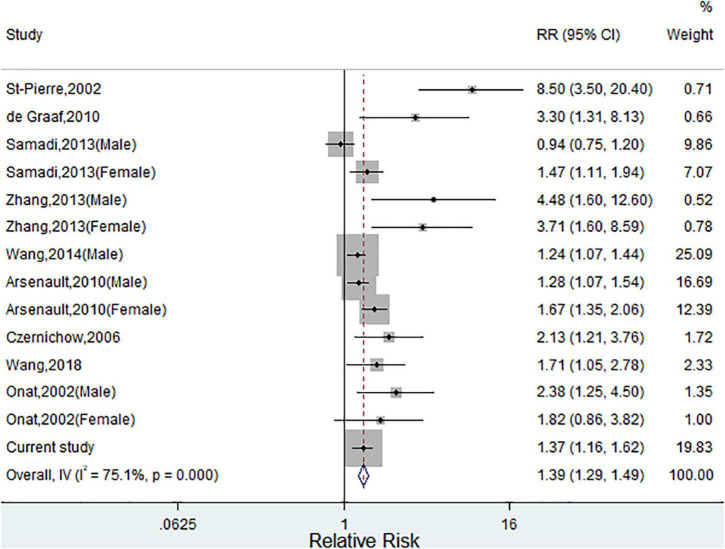
Meta-analysis of relative risks (RRs) and 95% CIs for the association between hypertriglyceridemic-waist phenotype and cardiovascular disease (CVD). (Squares represent weights; Horizontal lines represent confidence intervals; Diamond represent the merging effect value; Vertical line represents the invalid line).

Supplementary Figure 1 illustrates the study selection process in the meta-analysis. Finally, a total of nine published studies met the inclusion criteria and were included in the present meta-analysis with our current study (Supplementary Table 2) (18–24, 31, 32). The detailed characteristics of eligible studies were listed in Supplementary Table 1. Figure 2 shows the study-specific RRs and the pooled risk estimates. Consistent with our findings, this meta-analysis showed that individuals with HTGW phenotype were positively associated with CVD risk (*RRs* 1.39, 95% *CIs* 1.29–1.49) with significant heterogeneity (*I*^2^ = 75.1%, *p* = 0.007). The review of the funnel plot could not eliminate the potential publication bias (Supplementary Figure 3). Begg’s and Egger’s tests further suggested no evidence of potential publication bias (*p* > 0.05 for both). When sensitivity analyses were conducted according to sex, geographic area, and study design, there was a significant association between HTGW phenotype and CVD. Furthermore, subgroup analysis showed that the pooled risk estimates were significant in both coronary artery disease (*RRs* 2.25, 95% *CIs* 1.68–3.01) and stroke (*RRs* 1.98, 95% *CIs* 1.41–2.88) (Supplementary Table 3).

## Discussion

In this cohort study of Chinese aged 45 years and above among the CHARLS participants, we found that both NTGW and HTGW phenotypes were significantly associated with increased risk of CVD, stroke, and cardiac events over a 6-year follow-up period. In the meta-analysis based on available data from 9 previously published studies and the current study, we also found that individuals with HTGW phenotype had an increased risk of CVD.

The current findings on the significant association between HTGW phenotype and CVD were consistent with several previously published studies. For example, a cross-sectional study conducted in the Netherlands indicated that individuals with increased TG levels and WC showed a significantly elevated risk of any CAD (*ORs* 3.3, 95% *CIs* 1.31–8.13) and obstructive CAD (*ORs* 2.90, 95% *CIs* 1.16–7.28) (21). Arsenault et al. found that participants with HTGW phenotype were associated with a deteriorated cardiometabolic risk profile and an increased risk for CAD among European (31). A cohort study including 95,015 Chinese participants showed that both NTGW and HTGW phenotypes were associated with the increased risk of CVD, myocardial infarction, and ischemic stroke after a mean follow-up of 4 years (24). Nevertheless, inconsistent findings were also reported. In the above-reported cohort study of Chinese, no significant relationship was found between HTGW phenotype and hemorrhagic stroke (24). A cross-sectional study incorporating 2,296 Turkey adults indicated that the HTGW phenotype has predictability for CAD risk among men but not for women (32). While another cohort study of 6,834 participants showed that HTGW phenotype was the point of divergence for the prediction of CVD only among women (23).

To sum up, there are some obvious weak points of the previously reported studies, especially in different TG and WC cut-off values, study design, and insufficient adjustment, which may induce some bias and limit statistical power to evaluate the predictive value of HTGW phenotype on CVD. In the present study, we used longitudinal data from the CHARLS cohort study, which may have more power to determine the causal association between the HTGW phenotype and CVD risk. Also, the cut-off value of WC and TG was consistent with most of the previous studies and considered an appropriate cut-off in Chinese for central obesity and metabolic syndrome. Furthermore, age, sex, and several variables were adjusted in the multivariate Cox proportional hazards model, which reduced the influence of those confounding factors and improved the credibility of the findings. Therefore, the different results across the previously reported studies were plausible and intelligible.

Meta-analysis aggregate and quantify results from different studies to enhance statistical capabilities and provide more accurate and reliable risk estimates. In the present meta-analysis, we pooled published nine studies and the current study and further confirmed that the HTGW phenotype was associated with the CVD risk. The subgroup analysis showed that the pooled risk estimates were significant in both men and women, in Asia and non-Asia areas, and in different study designs. Taking together, all of those findings suggested that the HTGW phenotype may be a valuable predictor of CVD.

The previous studies verified that the HTGW phenotype could be used as an inexpensive screening tool to identify individuals characterized by the atherogenic metabolic triad (hyperinsulinemia, elevated apolipoprotein B, and small dense LDL-C particles) (7, 27). Furthermore, the predictive value of HTGW phenotype in hypertension (33), hyperuricemia (34), diabetes (35), and chronic kidney disease (35) has been verified in several studies, which were well-known risk factors. In the current study and meta-analysis, we further expanded and verified the prediction ability of the HTGW phenotype in CVD. Thus, it is of clinical interest for further study to test the application of the HTGW phenotype in clinical practice and other diseases.

The exact mechanisms underlying the association between HTGW phenotype and CVD remain incompletely elucidated. HTGW has been considered a precise marker of visceral obesity (14). Pieces of evidence had proved that visceral adiposity was more closely related to insulin resistance, hyperuricemia, diabetes, and hypertension, which were significantly associated with increased CVD risk (33–35). Furthermore, the HTGW phenotype was significantly linked with inflammation and adiponectin (35). Therefore, we observed the significant associations between HTGW and CVD are biologically plausible to a certain extent.

The present study was based on the data from the CHARLS, which is a large nationally representative cohort study in China with a high response rate. Moreover, various stratified analyses and adjustments for several established confounders also increase the credibility of the results. Some limitations should also be taken into consideration. First, although participants were included from CHARLS using a multistage probability sampling method, the present study was exclusively a Chinese population aged more than 45 years, and some proportion was excluded due to missing data or loss of follow-up, thus findings from our study might not be generalizable to other populations. However, the meta-analysis also verified previous and our findings. Second, although we carefully adjusted for several important confounders using various statistical models, we cannot exclude the potential residual confounding. Third, owing to the observational study design, a clear causal relation between HTGW phenotype and risk of CVD may not be established.

## Conclusion

Our results indicated that participants with NTGW or HTGW phenotypes were at an increased risk of developing CVD among Chinese adults aged 45 years and above. A meta-analysis also confirmed the significant findings. Our study suggested that simultaneous measurement of TG and WC might be a useful screening tool to identify high-risk individuals for developing CVD. The well-designed experimental research and prospective clinical studies are certainly warranted to confirm our findings.

## Data availability statement

The original contributions presented in the study are included in the article/Supplementary material, further inquiries can be directed to the corresponding author.

## Ethics statement

The ethics application for collecting data on human subjects in CHARLS was approved by the Biomedical Ethics Review Committee of Peking University (IRB00001052-11015). The patients/participants provided written informed consent to participate in this study.

## Author contributions

XZ and XR conceived and designed the research. XR wrote the manuscript. XR, MJ, and LH performed the data analysis. All authors reviewed the manuscript.

## Footnotes

1
http://charls.pku.edu.cn/en
