# Supplementary material for: Association between hypertriglyceridemic-waist phenotype and cardiovascular disease: A cohort study and meta-analysis
Source: Front Cardiovasc Med. 2022 Aug 4;9:940168. doi: 10.3389/fcvm.2022.940168 (PMC9386422; doi:10.3389/fcvm.2022.940168)
Supplement: Supplementary file 1 [file Data_Sheet_1.docx]

**Data S1**

**Supplemental Methods**

**Meta-analysis**

Based on the guidelines of the Preferred Reporting Items for Systematic reviews and Meta-Analyses (PRISMA)^1^, we conducted a meta-analysis to incorporate the results of the current study and previous published studies on the association of hypertriglyceridemic‑waist (HTGW) phenotype and cardiovascular disease (CVD). A systematic literature search was conducted on Embase, PubMed, Medline, and Web of Science from inception to April 1, 2022, using the search terms ‘hypertriglyceridemic waist’ (or ‘HTGW’, ‘NWNT’, ‘HTNW’, ‘NTGW’, ‘hypertriglyceridemic’) in combined with ‘cardiovascular disease’ (or ‘CVD’, ‘stroke’, ‘coronary artery disease’, ‘CAD’, ‘ischemic heart disease’, ‘hemorrhagic stroke’ and ‘myocardial infarction’), and the detailed search strategy was referred to previously published research (such as Table S1 in Wu et al study)^2^.We restricted the search to studies on humans that without any restrictions on language or study type. Reference lists of retrieved articles were manually scanned for all relevant additional studies and review articles. Studies were selected if they met following criteria: (1) included an investigation of the association between HTGW phenotype and CVD; (2) study design was a cross-sectional study, prospective study or case-control study; (3) reported covariate adjusted hazard ratios (HRs), relative risks (RRs), odds ratios (ORs) with 95% confidence intervals (CIs).

Literature search and data extraction were independently performed by two investigators. The following data elements were extracted from each included study: first author, publication year, country, study design, sample size, percentage of males, mean age, cut-off values of suboptimal waist circumference and triglycerides, outcome definition, adjusted covariates, and risk estimates of the association (HRs, ORs or RRs). Any disagreements were resolved by consensus. RRs was used as the effect size of the association between HTGW phenotype and CVD, and the ORs and HRs reported in the original studies were considered equivalent to RRs^3-4^

We examined heterogeneity in results across studies by using Cochrane’s Q test^5^and I^2^ statistic. Heterogeneity was present if the *P* value of the Q test was <0.1 or I^2^ >25%. If heterogeneity existed, the random effects model was used, otherwise, the fixed effects model was used. To account for potential bias, sensitivity analyses were performed according to sex, geographic area, study design and study outcome. Potential publication bias was examined using funnel plot, Begg test^6^and Egger’s asymmetry test^7^ All *P* values were two-sided, and the level of significance was at <0.05. All analyses were performed using STATA 14.0 (Stata Corp LP, College Station, Texas, USA).

**Supplemental Results**

**Meta-analysis**

Nine^8-16^published prospective studies and current study were included in this meta-analysis (Figure S1). The detailed characteristics of included studies are summarized in Table S1. All studies were published between 2002 and 2018. Five^10-12,15-16^ studies were conducted in Asia, there in Europe^9,13,14^ and one in the North America^8^.

Consistent with our findings, this meta-analysis indicated that the HTGW phenotype was significantly associated with increased risk of CVD (the pooled RR from all ten studies was 1.39(95% CI, 1.29-1.49; Figure 2). Substantial heterogeneity was observed across studies (I^2^=75.1%, *P*<0.001), which was probably caused by sample size and differences in study design. Review of the funnel plot could not eliminate potential publication bias (Figure S2). Begg and Egger tests further suggested no evidence of potential publication bias (*P*>0.05 for both). Several sensitivity analyses according to sex, geographic area, study design and study outcome further confirmed the significant association between HTGW phenotype and CVD (Table S2).

**Reference**

1. Liberati A, Altman DG, Tetzlaff J, et al. The PRISMA Statement for Reporting Systematic Reviews and Meta-Analyses of Studies That Evaluate Health Care Interventions: Explanation and Elaboration. Epidemiology Biostatistics & Public Health 2009;6:e1-e34.

2. Wu L, Sun D. Leptin Receptor Gene Polymorphism and the Risk of Cardiovascular Disease: A Systemic Review and Meta-Analysis. Int J Environ Res Public Health. 2017;14(4):375. Published 2017 Apr 3

3. Zhong C, Zhong X, Xu T, et al. Sex-Specific Relationship Between Serum Uric Acid and Risk of Stroke: A Dose-Response Meta-Analysis of Prospective Studies. J Am Heart Assoc 2017;6.

4. Yang WS, Va P, Wong MY, et al. Soy intake is associated with lower lung cancer risk: results from a meta-analysis of epidemiologic studies. American Journal of Clinical Nutrition 2011;94:1575-83.

5. Higgins JP, Thompson SG. Quantifying heterogeneity in a meta-analysis. Stat Med 2002;21:1539-58.

6. Begg C, Mazumdar M. Operating Characteristics of a Rank Correlation Test for Publication Bias. Biometrics 1995;50:1088-101.

7. Egger M. Language bias in randomised controlled trials published in English and German. Lancet 1997;350:326-9.

8. St-Pierre J, Lemieux I, Vohl MC, et al. Contribution of abdominal obesity and hypertriglyceridemia to impaired fasting glucose and coronary artery disease. Am J Cardiol. 2002;90(1):15-18.

9. de Graaf FR, Schuijf JD, Scholte AJ, et al. Usefulness of hypertriglyceridemic waist phenotype in type 2 diabetes mellitus to predict the presence of coronary artery disease as assessed by computed tomographic coronary angiography. Am J Cardiol. 2010;106(12):1747-1753.

10. Samadi S, Bozorgmanesh M, Khalili D, et al. Hypertriglyceridemic waist: the point of divergence for prediction of CVD vs. mortality: Tehran Lipid and Glucose Study. Int J Cardiol. 2013;165(2):260-265.

11. Zhang X, Shu XO, Li H, et al. Visceral adiposity and risk of coronary heart disease in relatively lean Chinese adults. Int J Cardiol. 2013;168(3):2141-2145.

12. Wang A, Li Z, Zhou Y, et al. Hypertriglyceridemic waist phenotype and risk of cardiovascular diseases in China: results from the Kailuan Study. Int J Cardiol. 2014;174(1):106-109.

13. Arsenault BJ, Lemieux I, Després JP, et al. The hypertriglyceridemic-waist phenotype and the risk of coronary artery disease: results from the EPIC-Norfolk prospective population study. CMAJ. 2010;182(13):1427-1432.

14. Czernichow S, Bruckert E, Bertrais S, Galan P, Hercberg S, Oppert JM. Hypertriglyceridemic waist and 7.5-year prospective risk of cardiovascular disease in asymptomatic middle-aged men. Int J Obes (Lond). 2007;31(5):791-796.

15. Wang W, Shen C, Zhao H, et al. A prospective study of the hypertriglyceridemic waist phenotype and risk of incident ischemic stroke in a Chinese rural population. Acta Neurol Scand. 2018;138(2):156-162.

16. Onat A, Ceyhan K, Başar O, Erer B, Toprak S, Sansoy V. Metabolic syndrome: major impact on coronary risk in a population with low cholesterol levels--a prospective and cross-sectional evaluation. Atherosclerosis. 2002;165(2):285-292.

**Supplemental Table S1. Baseline characteristics between the included and excluded groups.**

| Characteristics | Included |  | Excluded |  | *P* value |
| --- | --- | --- | --- | --- | --- |
| No. of subjects | 8216 |  | 9008 |  |  |
| Age, years | 58.91±9.39 |  | 59.34±10.31 |  | 0.052 |
| Sex, n (%) |  |  |  |  |  |
| Male | 3894(47.40) |  | 4503(49.99) |  | 0.007 |
| Female | 4322(52.60) |  | 4505(50.01) |  |  |
| Living place, n (%) |  |  |  |  |  |
| Urban | 2787(33.92) |  | 4180(46.40) |  | <0.001 |
| Rural | 5429(66.08) |  | 4828(53.60) |  |  |
| Education level, n (%) |  |  |  |  |  |
| Illiteracy | 2477(30.15) |  | 2291(25.43) |  | <0.001 |
| Primary school | 3346(40.73) |  | 3362(37.32) |  |  |
| Middle school | 1603(19.51) |  | 1949(21.62) |  |  |
| High school or above | 790(9.62) |  | 1406(15.61) |  |  |
| Medical history |  |  |  |  |  |
| Hypertension, n (%) | 1760(21.42) |  | 1939(21.53) |  | 0.869 |
| Dyslipidemia, n (%) | 622(7.57) |  | 1052(11.68) |  | <0.001 |
| Diabetes mellitus, n (%) | 445(5.42) |  | 654(7.26) |  | <0.001 |
| Smoking, n (%) | 3241(39.45) |  | 3626(40.25) |  | 0.075 |
| Drinking, n (%) | 3266(39.75) |  | 3467(38.49) |  | 0.352 |
| BMI (kg/m^2^) | 23.06(20.79-25.55) |  | 23.18(20.77-25.90) |  | 0.234 |
| TG, mg/dL | 104.43(74.34-151.34) |  | 112.40(80.54-169.04) |  | <0.001 |
| TC, mg/dL | 190.59(167.01-215.34) |  | 189.82(166.24-215.72) |  | 0.728 |
| LDL-C, mg/dL | 114.05(93.17-136.86) |  | 114.82(92.78-137.63) |  | 0.596 |
| HDL-C, mg/dL | 49.48(40.59-60.31) |  | 47.16(38.27-57.99) |  | <0.001 |
| FBG, mg/dL | 102.42(94.32-113.22) |  | 103.14(95.04-115.56) |  | 0.008 |
| SBP, mmHg | 135.11±11.87 |  | 138.46±9.71 |  | 0.377 |
| DBP, mmHg | 76.39±12.53 |  | 77.53±12.99 |  | 0.674 |

BMI: body mass index; TG: triacylglycerol; TC: total cholesterol; LDL-C: low-density lipoprotein-cholesterol; HDL-C: High-density lipoprotein-cholesterol; FBG: fasting blood glucose; SBP: systolic blood pressure; DBP: diastolic blood pressure;

Continuous variables are expressed as mean± standard deviation, or as median (interquartile range). Categorical variables are expressed as frequency (percent).

**Supplementary Table S2. Characteristics of the studies included in meta-analysis**

| **Author, year** | **Country** | **Study design** | **Sample size** | **Male (%)** | **Age (years)** | **WC cut-off (cm)** | | **TG cut-off (mmol/L)** | | **Disease outcomes** | **Quality** |
| --- | --- | --- | --- | --- | --- | --- | --- | --- | --- | --- | --- |
|  |  |  |  |  |  | **M** | **F** | **M** | **F** |  |  |
| St-Pierre,2002 ^8^ | Canada | Cross-sectional study | 569 | 100 | - | 90 |  | 2.0 |  | Coronary artery disease | 6 |
| de Graaf,2010^9^ | Netherlands | Cross-sectional study | 202 | 59.41 | 54±11 | 102 | 88 | 1.7 | 1.7 | Coronary artery disease | 6 |
| Samadi,2013^10^ | Iran | Cohort study  (median follow-up of 9.3 years) | 6,834 | 56.04 | M without HTGW  (49.15 (13.99))  M with HTGW  (49.46 (12.51))  F without HTGW  (45.65 (11.91))  F with HTGW  (51.90 (10.78)) | 86 | 91 | 1.8 | 2.0 | Cardiovascular diseases | 8 |
| Zhang,2013^11^ | China | Case-control study | 1,052 | 44.11 | 62.5±7.0 in female case  62.2±7.0 in female control  63.2±8.6 in male case  63.3±8.6 in male control | 90 | 85 | 2 | 1.5 | Coronary heart disease | 7 |
| Wang,2014^12^ | China | Cohort study  (median follow-up of 4 years) | 95,015 | 79.57 | aged 18–98 years | 90 | 85 | 2.0 | 1.5 | Cardiovascular disease  Ischemic stroke  Hemorrhagic stroke | 8 |
| Arsenault,2010^13^ | United Kingdom | Nested case–control | 2,840 | 62.96 | aged 45–79 years | 90 | 85 | 2 | 1.5 | Coronary artery disease | 7 |
| Czernichow,2006^14^ | France | Cohort study  (median follow-up of 7.5 years) | 3,430 | 100 | 51.8±4.7 in adult without CVD  54.0±4.8 in adult with CVD | 90 | - | 2 | - | Cardiovascular diseases | 8 |
| Wang,2018^15^ | China | Cohort study  (median follow-up of 5.16 years) | 4,081 | 40.60 | aged 35-75 years | 90 | 85 | 2 | 1.5 | Ischemic stroke | 8 |
| Onat,2002^16^ | Turkey | Cross-sectional study | 2,296 | 49.26 | 51.19±12.5 years | 90 | 92 | 1.58 | 1.36 | Coronary artery disease | 6 |

WC, waist circumference; TG, triglycerides; HTGW, high triglyceride levels and enlarged waist circumference; M, male; F, female;

**Supplementary Table S2. Characteristics of the studies included in meta-analysis (continue)**

| **Author, year** | **Covariates in main multivariate model** | **OR/HR (95%CI) in main multivariate model** | | | |
| --- | --- | --- | --- | --- | --- |
|  |  | **NTNW** | **HTNW** | **NTGW** | **HTGW** |
| St-Pierre,2002 ^1^ | age | Ref | 5.4(3.1-9.3) | 1.6(0.4-5.8) | 8.5(3.5-20.4) |
| de Graaf,2010^2^ | age, gender, obesity, hypertension, smoking, and family history of coronary artery disease | Ref | 1.5(0.58-3.95) | 1.1(0.48-2.71) | 3.3(1.31-8.13) |
| Samadi,2013^3^ | age, systolic blood pressure, using blood pressure lowering agents, total and high-density lipoprotein cholesterol, smoking,  and diabetes | Ref(M)  Ref(F) | - | - | 0.945(0.746-1.198)  1.470(1.111-1.944) |
| Zhang,2013^4^ | Age, total cholesterol, LDL cholesterol, HDL cholesterol, and non-HDL cholesterol | Ref(M)  Ref(F) | 2.21(0.96–5.08)  1.77(0.90–3.48) | 2.43(0.89–6.64)  1.99(0.71–5.55) | 4.48(1.60–12.6) (M)  3.71(1.60–8.59) (F) |
| Wang,2014^5^ | age and sex, average monthly income of each family member, education level, marital status, smoking status, drinking status, and physical  activity, hospitals, and adjusted for history of hypertension and diabetes mellitus, body mass index, total cholesterol level, low-density lipoprotein cholesterol level, high-density cholesterol level and high-sensitivity C-reactive | Ref(Cardiovascular disease)  Ref(Ischemic stroke)  Ref(Hemorrhagic stroke) | 1.13(0.96–1.34)  1.08(0.87–1.34)  1.09(0.78–1.52) | 1.15(1.02–1.30)  1.18(1.01–1.38)  1.06(0.82–1.38) | 1.24(1.07–1.44)  1.23(1.01–1.49)  0.97(0.80–1.33) |
| Arsenault,2010^6^ | age, total cholesterol level, high-density lipoprotein cholesterol level, systolic blood pressure, smoking status and presence of diabetes | Ref  Ref | 1.12(0.84–1.48  1.35(1.09–1.67) | 1.26(1.04–1.53)  1.34(1.04–1.73) | 1.28(1.07–1.54)  1.67(1.35–2.06) |
| Czernichow,2006^7^ | age, active smoking, physical activity, SBP, DBP and fasting blood glucose | Ref | 0.72(0.17-3.05) | 1.22(0.76-1.95) | 2.13(1.21-3.76) |
| Wang,2018^8^ | age, gender, smoking status, drinking status, history  of diabetes mellitus, history of hypertension, total cholesterol level, high-density lipoprotein cholesterol level, and low-density lipoprotein cholesterol level | Ref | 1.26(0.70-2.29) | 1.36(0.85-2.16) | 1.71(1.05-2.78) |
| Onat,2002^9^ | age | Ref(M)  Ref(F) | 0.69(NS)  1.28(NS) | 2.26(1.08-4.30)  1.82(0.84-3.93) | 2.38(1.25-4.50)  1.82(0.86-3.82) |

M, male; F, female; OR, odds ratio; HR, hazard ratio; NTNW: normal triglyceride levels and normal waist circumference; HTNW: high triglyceride levels and normal waist circumference; NTGW: normal triglyceride levels with enlarged waist circumference; HTGW: high triglyceride levels and enlarged waist circumference.

**Supplemental Table S3**. **Meta-analyses of hypertriglyceridemic-waist phenotype and CVD: sensitivity analyses**

|  | N | RRs (95% CIs) | *P* value |
| --- | --- | --- | --- |
| Total | 10 | 2.24(1.70-2.95) | <0.001 |
| Sex |  |  |  |
| Male | 6 | 1.20(1.06-1.35) | 0.002 |
| Female | 4 | 1.39(1.20-1.61) | 0.008 |
| Geographic area |  |  |  |
| Asia | 5 | 1.29(1.18-1.418) | <0.001 |
| Non-Asia | 5 | 1.48(1.25-1.75) | <0.001 |
| Study design |  |  |  |
| Cross-sectional study | 3 | 3.00(2.04-4.41) | 0.005 |
| Case-control study | 2 | 1.50(1.31-1.71) | 0.016 |
| Cohort study | 5 | 1.28(1.17-1.40) | 0.008 |
| Study outcome |  |  |  |
| Coronary artery disease | 6 | 2.25(1.68-3.01) | 0.003 |
| Stroke | 4 | 1.98(1.41-2.88) | <0.001 |

RRs, relative risks; CIs, confidence intervals.

**Figure S1.** Flow chart of study selection of meta-analysis.


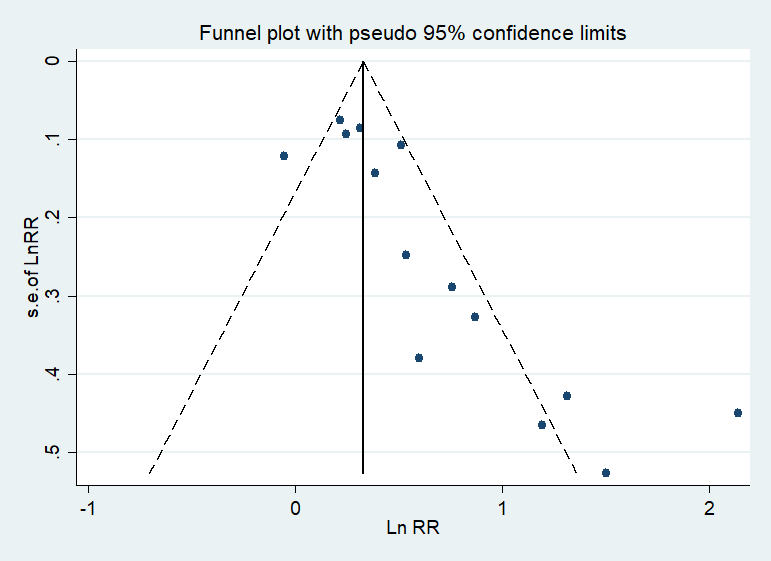


**Figure S2.** Funnel plot of hypertriglyceridemic-waist phenotype and CVD.
